# Pharmacokinetic Cross-Over Study of Pharmacy-Compounded Chenodeoxycholic Acid Capsules Compared to Authorized Capsules

**DOI:** 10.3390/pharmaceutics17121525

**Published:** 2025-11-27

**Authors:** Natalja Bouwhuis, Bart A. W. Jacobs, Soumia Majait, Frédéric M. Vaz, Carla E. M. Hollak, Eleonora L. Swart, E. Marleen Kemper

**Affiliations:** 1 Department of Pharmacy and Clinical Pharmacology, Amsterdam UMC, Meibergdreef 9, 1105 AZ Amsterdam, The Netherlands; b.jacobs@nki.nl (B.A.W.J.); s.majait@amsterdamumc.nl (S.M.); el.swart@amsterdamumc.nl (E.L.S.); m.kemper@apotheeka15.nl (E.M.K.); 2 Platform Medicine for Society, Amsterdam UMC, Meibergdreef 9, 1105 AZ Amsterdam, The Netherlands; c.e.hollak@amsterdamumc.nl; 3 Laboratory Genetic Metabolic Diseases, Department of Clinical Chemistry and Pediatrics, Emma Children’s Hospital, Amsterdam UMC, Meibergdreef 9, 1105 AZ Amsterdam, The Netherlands; f.m.vaz@amsterdamumc.nl; 4 Amsterdam Gastroenterology Endocrinology Metabolism, Inborn Errors of Metabolism, Meibergdreef 9, 1105 AZ Amsterdam, The Netherlands; 5 Core Facility Metabolomics, Amsterdam UMC, Meibergdreef 9, 1105 AZ Amsterdam, The Netherlands; 6 Department of Endocrinology and Metabolism, Amsterdam UMC, Meibergdreef 9, 1105 AZ Amsterdam, The Netherlands

**Keywords:** chenodeoxycholic acid, pharmacokinetics, pharmacy compounding, cerebrotendinous xanthomatosis, randomized clinical trial

## Abstract

**Purpose:** The Amsterdam UMC pharmacy has been compounding chenodeoxycholic acid (CDCA) capsules for Dutch cerebrotendinous xanthomatosis patients since 2018. However, limited data are available on the pharmacokinetics and bioequivalence of therapeutic CDCA formulations. **Methods:** An open-label, single-center, randomized, two-period, two-sequence, cross-over study was conducted in 12 healthy volunteers to compare the pharmacokinetic profile of pharmacy-compounded CDCA capsules to that of the authorized CDCA product. **Results:** Both formulations reached peak plasma concentrations (t_max_) at approximately 1 h post-dose. The mean AUC_(0–6h)_ values were 262.4 (±69.4) µmol∙min/L for the compounded capsules and 248.0 (±78.1) µmol∙min/L for the authorized capsules, with a 90% confidence interval (CI) for the AUC_(0–6h)_ ratio of 0.89–1.30, exceeding the accepted bioequivalence range of 0.80–1.25. The mean C_max_ for the compounded formulation (2.96 ± 0.91 µmol/L) was significantly lower than that of the comparator product (4.42 ± 1.36 µmol/L; *p* = 0.0040), with a 90% CI for the C_max_ ratio of 0.57–0.80, also outside the bioequivalence range. **Conclusions:** Overall, the pharmacy-compounded and authorized capsules demonstrate a comparable AUC_(0–6h)_ and t_max_. Bioequivalence could not be demonstrated, primarily due to high variation, a significantly lower C_max_, and an AUC_(0–6h)_ ratio outside the accepted limits. These findings indicate that the compounded formulation results in reduced systemic peak exposure compared with the authorized product. However, given the high variation, a larger sample size would be needed to further investigate bioequivalence in future studies.

## 1. Introduction

Chenodeoxycholic acid (CDCA) is used therapeutically in the treatment of cerebrotendinous xanthomatosis (CTX), a rare genetic inborn error of bile acid synthesis. CDCA is one of the primary endogenous bile acids and is synthesized in the liver from cholesterol via two major bile acid synthesis pathways. The classic or neutral pathway that accounts for approximately 90% of bile acid synthesis starts with hydroxylation of cholesterol by CYP7A1 to form 7α-hydroxycholesterol, followed by formation of CDCA via sterol 27-hydroxylase (CYP27A1) [1,2]. The alternative pathway that accounts for approximately 10% of bile acid synthesis starts with hydroxylation of cholesterol by CYP27A1 to 27-hydroxycholesterol followed by conversion to CDCA via CYP7B1 [1,2]. CDCA inhibits CYP7A1, establishing a negative feedback loop that inhibits bile acid synthesis when there are sufficient bile acids [1]. CTX is characterized by CYP27A1 deficiency, and as CYP27A1 is essential in CDCA synthesis, CTX patients have severely decreased levels of endogenous CDCA, stimulating increased production of bile acids and the generation and accumulation of atypical sterol molecules including bile alcohols (and their corresponding glucoronides) and cholestanol [3]. This leads to various severe disease symptoms such as infantile-onset diarrhea, juvenile cataract and tendon xanthomas, as well as adult onset of neurologic dysfunction [3]. Treatment with CDCA is used as replacement for endogenous CDCA that silences the bile acid synthetic pathway via downregulation of CYP7A1, which in turn can prevent the onset of neurological complications in CTX patients when initiated before neurological symptoms are present [4].

Limited information is available on the pharmacokinetics of therapeutic CDCA. Orally administered CDCA is absorbed in the small intestine, binds to plasma proteins, and is cleared by the liver [5,6]. In the liver, CDCA is almost completely conjugated with glycine or taurine before being excreted into bile and transported to the intestine and into the enterohepatic circulation along with endogenous bile acids [2,5,6]. Conjugated CDCA is reabsorbed in the jejunum and the terminal ileum [7]. However, reabsorption is not complete and is variable (29- 84%) [6]. Unabsorbed CDCA is metabolized by gut bacteria into ursodeoxycholic acid (UDCA) and lithocholic acid (LCA) [1,2]. These secondary bile acids are then mostly reabsorbed in the distal ileum, secreted into the portal circulation and transported back to the liver. This efficient enterohepatic circulation ensures that 95% of bile acids are returned to the liver and that only 5% of total bile acids, and also therapeutic bile acids, are excreted in the feces [1,2,5]. Because of the enterohepatic circulation, serum bile acid concentrations are low and the volume of distribution is high [5]. Upon supplementation of CDCA, peak serum concentrations are expected after 50–120 min. CDCA has a biological half-life of approximately 45 h to 4 days in the enterohepatic circulation [5,6,7].

CDCA has been used off-label for treatment of CTX for many years. In 2017, Leadiant CDCA capsules received a marketing authorization in the EU as an orphan drug for the treatment of CTX (EU/1/16/1110) [6]. The price increase that followed and subsequent non-reimbursement under the Dutch healthcare insurance meant that Leadiant CDCA was no longer available for Dutch CTX patients. In order to continue patient care, the Amsterdam UMC hospital pharmacy has been compounding CDCA capsules as a magistral formula preparation for its own patients since 2018, which means that approximately 60 Dutch patients now receive these pharmacy-compounded CDCA capsules [8]. The pharmacy-compounded capsules are prepared manually using a dry powder blend of CDCA active pharmaceutical ingredient (API) and the single excipient silica (colloidal anhydrous). In comparison, the authorized product is produced automatically from granulated powder and contains the excipients maize starch, magnesium stearate, silica (colloidal anhydrous), and water [6].

As described, little to no clinical data can be found on the pharmacokinetics of therapeutic CDCA. To be able to receive a marketing authorization, pharmacokinetic studies are mandatory, but for pharmacy preparations, this is not the case. To obtain a clear picture of the pharmacokinetics of therapeutic CDCA, we performed this randomized cross-over clinical trial in which the pharmacokinetic characteristics of the pharmacy-compounded CDCA capsules were investigated and compared with that of authorized CDCA capsules. Additionally, a bioequivalence analysis was performed. Lastly, the impact of single-dose CDCA administration on the bile acid profile in serums and on safety parameters was evaluated.

## 2. Materials and Methods

### 2.1. Trial Registration

The trial is registered in the Netherlands Trial Register with registration number NL9736, and was approved by medical ethics committee Academisch Medisch Centrum Amsterdam on 1 November 2021 (Eudra-CT: 2021-003823-14).

### 2.2. Study Design

The study was conducted at the Amsterdam UMC hospital following Good Clinical Practices (GCP) guidelines. The study was an open-label, single-center, randomized, two-period, two-sequence, cross-over study in healthy volunteers, to compare the pharmacy-compounded CDCA capsules with the authorized CDCA capsules. The study was set up according to the EMA Guideline on the Investigation of Bioequivalence to also be able to conduct additional bioequivalence analysis [9]. A cross-over design was chosen as it allowed each subject to act as their own control, reducing variability. Subjects were randomly allocated (1:1) by block randomization (block size: 2) to one of the two study arms and received a single oral dose of 250 mg CDCA of both products, separated by a wash-out period of at least one week [9]. Baseline level determination of CDCA in plasma was not deemed necessary. Although it is an endogenous substance, normal (free) serum levels are extremely low as CDCA is mostly confined in the enterohepatic circulation.

### 2.3. Test Product and Comparator Product

The test product, pharmacy-compounded CDCA 250 mg hard capsules, were developed and manufactured by the Amsterdam UMC hospital pharmacy under Good Manufacturing Practices (GMP) conditions and are clear gelatine capsules filled with 250 mg CDCA API and 0.5% silica (colloidal anhydrous) as a lubricant. For more information on product development, formulation, validation, production and dissolution, we refer to our earlier publication [10]. Authorized CDCA Leadiant 250 mg hard capsules (EU/1/16/1110/001) were used as the comparator product [6].

### 2.4. Subjects

Healthy, non-smoking, non-medication-using, adult, male volunteers were included with a BMI between 18.5 and 30 kg/m^2^ and with no (history of) alcohol and/or drug abuse, gastrointestinal disease, metabolic or endocrine disease, liver disease, or gallbladder disease and/or removal. Subjects were not allowed to participate in another clinical study during or in the three months prior to the study. Informed consent was signed prior to the start of the study. Subjects were included until there were 12 evaluable subjects, according to the EMA Guideline on the Investigation of Bioequivalence. No formal sample size calculation was performed [9].

### 2.5. Clinical Protocol and PK Sampling

The EMA guideline recommends to standardize diet, fluid intake and exercise [9]. The study was conducted in fasting state (at least 8 h prior to administration) to minimize variability. When ingested with food, absorption may be delayed but bioavailability is unaltered [7]. Subjects needed to abstain from food and drinks which may interact with circulatory, gastrointestinal, hepatic or renal function (e.g., alcoholic drinks or certain fruit juices such as grapefruit juice), from 24 h before to 6 h post administration. To standardize exercise, subjects were not allowed to exercise strenuously in the 24 h before administration, defined as more than one hour of exercise. The capsules were taken whole with 200 mL of water. During the study, drinking of water was allowed as desired, except for one hour before and one hour after drug administration. Food was not allowed. Blood sampling was performed at t = 0, 15, 30, 45, 60, 90, 120, 180, 240 and 360 min.

### 2.6. Bioanalytical Analysis

Serum CDCA, CA, DCA, LCA and UDCA levels were determined in free form and in conjugated form with LC-MS/MS by the ISO 15189:2012 certified laboratory of Genetic Metabolic Diseases of the Amsterdam UMC, essentially as described previously [11].

### 2.7. Pharmacokinetic Analysis and Statistics

Analysis was performed on results of bile acids in free form, as this is expected to be more sensitive for detecting differences in bioavailability. To determine and compare the pharmacokinetic profile, the AUC_(0–6h)_, AUC_(0–∞)_, residual area, C_max_ and t_max_ were determined using the Microsoft Excel add-in PKSolver non-compartmental analysis (linear up/log down, 4 points terminal slope). No cross-validation of results was performed. For statistical analysis and data visualization, GraphPad Prism 10.2.0 was used. Differences between the groups were tested with a paired parametric *t*-test (two-tailed *p* < 0.05). To investigate bioequivalence, AUC_(0–6h)_ and C_max_ were evaluated using RStudio version 2023.12.1 following the “nlme” package method as described by Park et al. [12]. For these parameters, the 90% confidence interval for the ratio of the test product and comparator should be contained within the acceptance interval of 80.00–125.00% to study bioequivalence.

### 2.8. Side Effects

Subjects were asked to report any experienced side effects and these were evaluated by the responsible physician to determine if they could be related to the treatment.

## 3. Results

### 3.1. Subject Demographics

In total 12 subjects were included, all of whom completed the study. Subject demographics are shown in Table 1.

### 3.2. Pharmacokinetics

**Figure 1:**
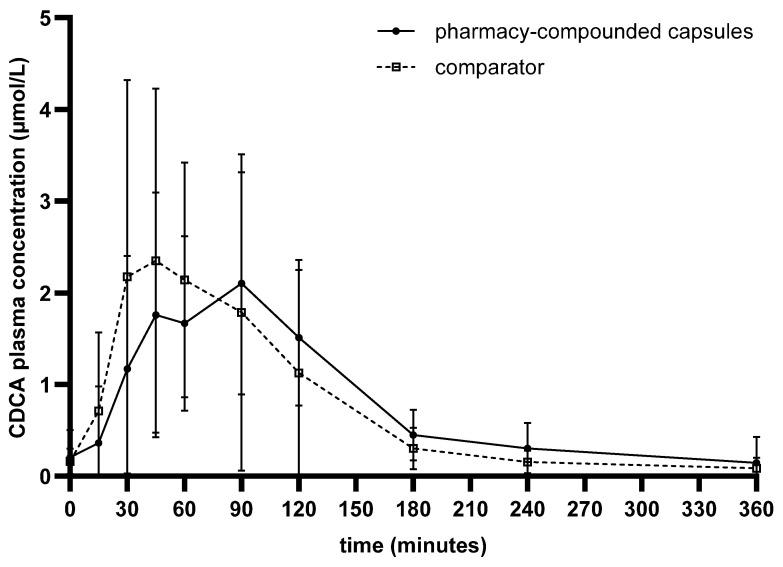
Concentration–time curve of free CDCA in plasma, average of 12 subjects for each treatment. Standard deviation (SD) as error bars.

**Figure A1:**
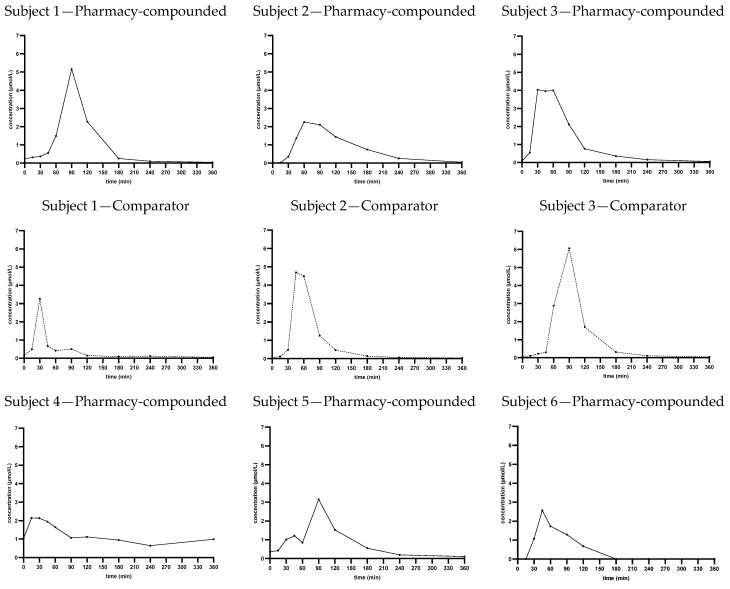

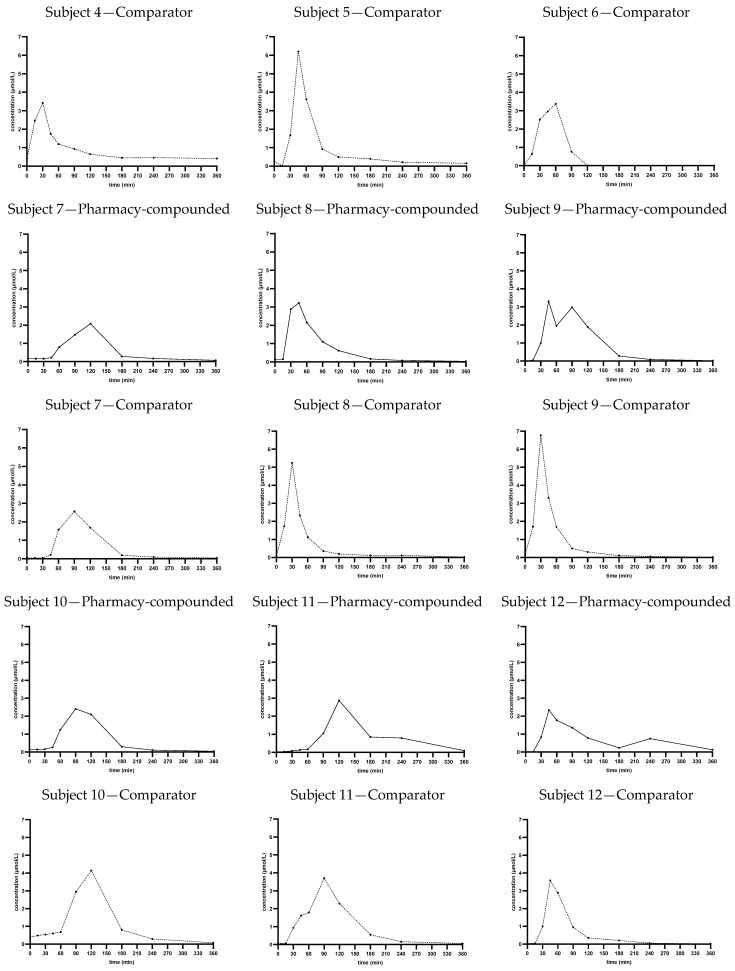
Individual concentration time curves of free CDCA in plasma over time of all 12 subjects and both treatments.

The average concentration time curves are shown in Figure 1. Individual concentration time curves of all 12 subjects on both treatments are shown in Appendix A Figure A1. The pharmacokinetic parameters AUC_(0–6h)_, C_max_ and t_max_ and their respective mean, standard deviation, range and 95% CI are calculated and shown in Table 2.

**Figure 2:**
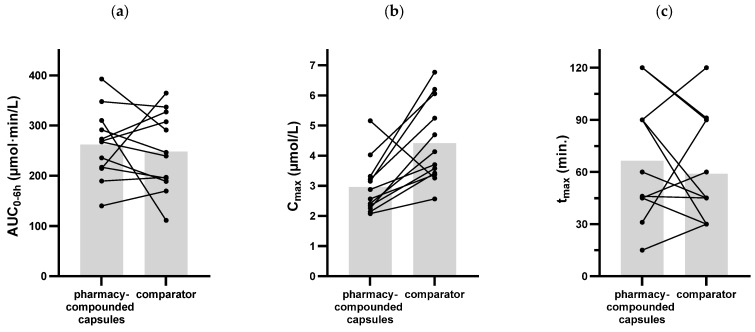
AUC_(0–6h)_ (**a**), C_max_ (**b**) and t_max_ (**c**) for individual subjects, pharmacy-compounded capsules vs. comparator product. Means shown as bars.

The average AUC_(0–6h)_ for the compounded capsules and the comparator product was 262.4 µmol∙min/L and 248.0 µmol∙min/L, respectively. A visualization of all individual AUC_(0–6h)_ values per subject is shown in Figure 2a. The average C_max_ for the compounded capsules was 2.96 µmol/L and for the comparator product 4.42 µmol/L. Individual C_max_ values are shown in Figure 2b. The average C_max_ is calculated by taking the average of all individual C_max_ values and is therefore different from the peak visually observed in the average concentration–time curve in Figure 1 but is clearly visible in Figure 2b. The average t_max_ for the compounded capsules was 66.4 min and for the comparator product 58.6 min. Individual t_max_ values are shown in Figure 2c.

A *t*-test was used to compare the pharmacokinetic parameters of the two treatments and results are shown in Table 2. AUC_(0–6h)_ and t_max_ showed no significant difference. The average C_max_ of the comparator product was significantly higher than the C_max_ of the pharmacy-compounded capsules (*p* = 0.0040).

The average concentration measured at t = 0 for both treatment groups is 0.17 ± 0.22 µmol/L, and 0.11 ± 0.20 µmol/L at t = 6 h. This shows that there are endogenous baseline levels, but they are low compared to the measured C_max_. The average concentration after 6 h is similar to the t = 0 concentration, showing that the latest sampling moment was chosen well. This can also be observed from Figure 1. However, due to the large variation, especially in the elimination phase of the curves, and due to the minimal endogenous CDCA baseline levels, the AUC_(0–∞)_ and residual area could not be calculated as planned in a reliable way and therefore these results are not shown.

### 3.3. Bioequivalence Analysis

Results of bioequivalence analysis are shown in Table 3 and showed a 90% CI of 0.89–1.30 and 0.57–0.80 for AUC_(0–6h)_ and C_max_, respectively. The treatment had a significant effect (*p* = 0.0021) on C_max_.

### 3.4. Bile Acid Profile and Safety

**Figure A2:**
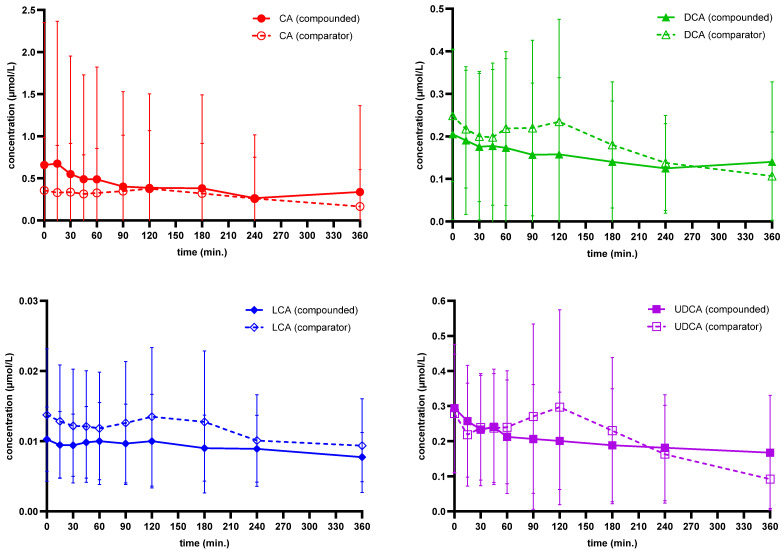
Concentration–time curves of CA, DCA, LCA and UDCA after oral administration of 250 mg CDCA, compounded capsules vs. comparator capsules. Average of 12 subjects. SD as error bars.

The concentration–time curves of free CA, DCA, LCA and UDCA after one-time oral administration of 250 mg CDCA are shown in Appendix A Figure A2. In one subject, high CA levels were observed, which contributed to the relatively high CA levels. There was not enough change in bile acid levels to reliably calculate AUCs and compare between the treatments.

One subject reported headache, dizziness and red rash (chest/neck) for 3 days after the pharmacy-compounded CDCA capsule was administered. This adverse event was evaluated by the responsible physician and judged as mildly severe and unlikely to be related to the treatment.

## 4. Discussion and Conclusions

With this study we provide the pharmacokinetic data of pharmacy-compounded CDCA capsules. As described in the introduction, limited research has been performed on the pharmacokinetics of therapeutic CDCA, and in the form of pharmacy-compounded capsules even less. Therefore, the results of this study provide a valuable insight into the pharmacokinetic profile of CDCA capsules. The pharmacokinetic parameters and the concentration–time curve of the capsules show that orally administered CDCA is well absorbed and further distributed and metabolized within 6 h. Comparing the pharmacy-compounded CDCA capsules to the authorized CDCA capsules, the average concentration–time curves for the two products appear similar and the average AUC_(0–6h)_ and t_max_ are not significantly different, indicating that the two products show the same exposure rate and have a similar total absorption and bioavailability. The average t_max_ is similar and around 1 h after administration.

The average C_max_, however, shows a high variability and is significantly higher in the comparator product than in the pharmacy-compounded capsules, suggesting a lower absorption rate of the compounded capsules. This difference in C_max_ is not observed looking at the average concentration–time curves in Figure 1, indicating also a high variation in t_max_ between individuals. This can be confirmed from the individual concentration–time curves in Appendix A Figure A1 and the plots in Figure 2. An explanation for the lower average C_max_ would require additional investigation. It could be related to the formulation of the capsules, as the dry powder mixture with limited excipients from the pharmacy-compounded capsules can affect absorption differently than the wet granulation production method of the comparator product, leading to a lower absorption rate. However, this is only speculative. It is also possible that with more sampling time points around t_max_, the C_max_ can be determined more accurately and less or no significant difference might be observed.

Both the concentration–time curves and the pharmacokinetic parameters of the two products show a high standard deviation and a broad range, indicating that there is a high inter-subject variability for therapeutic CDCA. Considerable inter- and intra-subject variability in peak serum concentrations makes bioavailability comparison difficult. A cross-over study design was chosen to eliminate the interindividual variability factor; however, a large standard deviation was still observed. This might be explained by the fact that CDCA is an endogenous bile acid with a complex metabolism that is mainly confined in the enterohepatic circulation, making plasma levels less clinically relevant. CTX patients have reduced CDCA levels, and therefore the therapeutic effectiveness of CDCA can better be related to the concentration in bile than to plasma concentrations [5,7]. Therefore, the lower average C_max_ of the compounded CDCA capsules is expected to have little clinical consequences. Most patients with CTX receive a chronic dose of 250 mg three times a day, making total exposure in the enterohepatic circulation more relevant than peak plasma concentrations. Also, as CDCA is an endogenous bile acid and individual C_max_ plasma values already vary widely between individuals, the therapeutic window is likely not that narrow. These factors mean that this study is not directly applicable to patients with CTX undergoing chronic treatment. This study focuses on the investigation and comparison of the pharmacokinetics after a single dose. Under controlled conditions such as this clinical study, the AUC_(0–6h)_ can be used for bioavailability comparison [7]. As the average AUC_(0–6h)_ is not significantly different between the treatments, we can conclude that the bioavailability of the two products is similar.

Additionally, bioequivalence analysis was performed to investigate if bioequivalence can be demonstrated based on the data from this study. Results show that the 90% confidence intervals of the ratio of the AUC_(0–6h)_ and the C_max_ of the pharmacy-compounded capsules and the comparator product are not within the acceptance criterium of 80.00–125.00% [9]. Even for AUC_(0–6h)_, which has a similar mean which is not significantly different, the confidence interval is too wide. This is probably due to the high variability as discussed earlier. A larger sample size would normally narrow the confidence interval if the AUCs_(0–6h)_ are indeed similar. Bioequivalence analysis did demonstrate a significant effect of the treatment on C_max_ and confirmed that the compounded capsules have a lower C_max_. A different study set-up would be required to properly investigate bioequivalence. However, that was not the goal of this study and the pharmacy-compounded capsules were not developed to be bioequivalent to the authorized product, but to provide our CTX patients with a qualitative CDCA treatment.

Lastly, the effect of a one-time oral dose of 250 mg CDCA on other bile acids and on side effects was investigated. Little changes are observed in other bile acids, which is also not to be expected after a single dose. One adverse event occurred during the study, but was evaluated as unrelated to the treatment.

To make this study feasible for a hospital pharmacy, various choices were made in the study set-up. Firstly, the study was performed with the minimum required number of healthy volunteers according to the EMA guideline for bioequivalence. However, in hindsight, since we found a high variability, a larger sample size would be desirable to narrow confidence intervals and thereby strengthen conclusions. Since little information was available on the pharmacokinetics of therapeutic CDCA, our results might be helpful in sample size calculations for future studies.

Also, no baseline correction was performed for endogenous plasma CDCA levels. Extremely low baseline levels in plasma were expected as CDCA is mostly confined in the enterohepatic circulation. If substantial increases over baseline endogenous levels are seen, baseline correction may not be needed. On average we found CDCA plasma levels at t = 0 of 4.6% of the average C_max_. Although the found endogenous levels are indeed low, performing a baseline correction might have strengthened our conclusions.

Furthermore, we chose to perform this study groups that were standardized as much as possible (fasted healthy male volunteers in a cross-over setting) to be able to compare the two treatments as well as possible in a small group. This resulted in exclusion of female participants, which is a limitation as sex-related pharmacokinetic differences were not investigated. For future studies, it would be interesting to also include females for potential sex impact, and to include subjects in fasted versus non-fasted state to investigate the effects of food. Lastly, to be able to translate results to clinical practice, it would be useful to perform a pharmacokinetic study in CTX patients.

In conclusion, the pharmacokinetic profile of the pharmacy-compounded CDCA capsules shows that the product is well absorbed and that there is high variability in absorption rate and C_max_ between individuals. The average AUC_(0–6h)_ is not significantly different between the compounded capsules compared to the registered product, indicating a similar extent of absorption.

## Figures and Tables

**Table 1:** Subject demographics (*n* = 12).

Characteristic	Mean ± SD (Range)
Age (years)	44.3 ± 20.3 (23–72)
Height (cm)	181.0 ± 11.3 (156–192)
Weight (kg)	77.6 ± 11.2 (54–92)
BMI (kg/m^2^)	23.5 ± 3.1 (18.8–29.7)

**Table 2:** Pharmacokinetic parameters of the pharmacy-compounded capsules vs. the comparator capsules. Average of 12 subjects.

	Pharmacy-Compounded CDCA Capsules			Comparator CDCA Capsules			*t*-Test
	Mean ± SD (Range)	CV	95% CI	Mean ± SD (Range)	CV	95% CI	*p* Value
AUC_(0–6h)_ (µmol∙min/L)	262.4 ± 69.4 (139.9–392.7)	26.4%	218.3–306.5	248.0 ± 78.1 (111.0–364.6)	31.5%	198.3–297.6	0.5726
C_max_ (µmol/L)	2.96 ± 0.91 (2.08–5.16)	30.6%	2.39–3.54	4.42 ± 1.36 (2.57–6.77)	30.8%	3.55–5.28	0.0040 **
t_max_ (min.)	66.4 ± 34.6 (15–120)	52.0%	44.5–88.4	58.6 ± 31.1 (30–120)	52.8%	39.1–78.6	0.4466

** statistically significant *p* < 0.01.

**Table 3:** Results of analysis on AUC_(0–6h)_ and C_max_ for evaluation of bioequivalence using R “nlme” method.

				Geometric Mean Ratio	
	Fixed Effects	(t Value)^2^	*p*-Value	Point Estimate	90% CI
AUC_(0–6h)_	Group	0.4497	0.5177	1.08	0.89–1.30
	Period	2.3793	0.1540		
	Treatment	0.5224	0.4864		
C_max_	Group	0.0688	0.7984	0.67	0.57–0.80
	Period	0.2128	0.6544		
	Treatment	16.8646	0.0021 **		

** statistically significant *p* < 0.01.

## Data Availability

Data available on request from the authors.

## References

[1] Fiorucci S., Distrutti E. (2019). Chenodeoxycholic Acid: An Update on Its Therapeutic Applications. Bile Acids and Their Receptors.

[2] Fiorucci S., Distrutti E. (2019). The Pharmacology of Bile Acids and Their Receptors. Bile Acids and Their Receptors.

[3] Federico A., Gallus G.N., Adam M.P., Bick S., Mirzaa G.M., Pagon R.A., Wallace S.E., Amemiya A. (2003). Cerebrotendinous Xanthomatosis. GeneReviews^®^ [Internet].

[4] Stelten B.M.L., Lycklama A.N.G.J., Hendriks E., Kluijtmans L.A.J., Wevers R.A., Verrips A. (2022). Long-term MRI Findings in Patients with Cerebrotendinous Xanthomatosis Treated With Chenodeoxycholic Acid. Neurology.

[5] Crosignani A., Setchell K.D., Invernizzi P., Larghi A., Rodrigues C.M., Podda M. (1996). Clinical pharmacokinetics of therapeutic bile acids. Clin. Pharmacokinet..

[6] Leadiant GmbH (2017). Chenodeoxycholic Acid Leadiant: EPAR—Product Information. https://www.ema.europa.eu/en/documents/product-information/chenodeoxycholic-acid-leadiant-epar-product-information_en.pdf.

[7] Iser J.H., Sali A. (1981). Chenodeoxycholic acid: A review of its pharmacological properties and therapeutic use. Drugs.

[8] Sheldon T. (2018). Dutch hospital makes own drug for rare condition after manufacturer hikes price to €170 000. BMJ.

[9] EMA (2010). Guideline on the Investigation of Bioequivalence (Rev.1).

[10] Bouwhuis N., Jacobs B.A.W., Kemper E.M. (2023). Product development and quality of pharmacy compounded chenodeoxycholic acid capsules for Dutch cerebrotendinous xanthomatosis patients. Front. Pharmacol..

[11] Bootsma A.H., Overmars H., van Rooij A., van Lint A.E., Wanders R.J., van Gennip A.H., Vreken P. (1999). Rapid analysis of conjugated bile acids in plasma using electrospray tandem mass spectrometry: Application for selective screening of peroxisomal disorders. J. Inherit. Metab. Dis..

[12] Park G., Kim H., Bae K.S. (2020). Bioequivalence data analysis. Transl. Clin. Pharmacol..

